# Myocarditis-associated necrotizing coronary vasculitis: incidence, cause, and outcome

**DOI:** 10.1093/eurheartj/ehaa973

**Published:** 2020-12-23

**Authors:** Andrea Frustaci, Maria Alfarano, Romina Verardo, Chiara Agrati, Rita Casetti, Fabio Miraldi, Nicola Galea, Claudio Letizia, Cristina Chimenti

**Affiliations:** Department of Clinical, Internal, Anesthesiologist and Cardiovascular Sciences, Sapienza University, Viale del Policlinico 155, 00161 Rome, Italy; Cellular and Molecular Cardiology Lab, IRCCS “Lazzaro Spallanzani”, Via Portuense, 292, 00149 Rome, Italy; Department of Clinical, Internal, Anesthesiologist and Cardiovascular Sciences, Sapienza University, Viale del Policlinico 155, 00161 Rome, Italy; Cellular and Molecular Cardiology Lab, IRCCS “Lazzaro Spallanzani”, Via Portuense, 292, 00149 Rome, Italy; Cellular Immunology Laboratory, IRCCS “Lazzaro Spallanzani”, Via Portuense, 292, 00149 Rome, Italy; Cellular Immunology Laboratory, IRCCS “Lazzaro Spallanzani”, Via Portuense, 292, 00149 Rome, Italy; Department of Clinical, Internal, Anesthesiologist and Cardiovascular Sciences, Sapienza University, Viale del Policlinico 155, 00161 Rome, Italy; Department of Experimental Medicine, Sapienza University, Viale del Policlinico 155, 00161 Rome, Italy; Department of Translation Medicine and Precision, Sapienza University, Viale del Policlinico 155, 00161 Rome, Italy; Department of Clinical, Internal, Anesthesiologist and Cardiovascular Sciences, Sapienza University, Viale del Policlinico 155, 00161 Rome, Italy; Cellular and Molecular Cardiology Lab, IRCCS “Lazzaro Spallanzani”, Via Portuense, 292, 00149 Rome, Italy

**Keywords:** Myocarditis, Vasculitis, Viral infection, Autoimmune disease

## Abstract

**Aims:**

Necrotizing coronary vasculitis (NCV) is a rare entity usually associated to myocarditis which incidence, cause, and response to therapy is unreported.

**Methods and results:**

Among 1916 patients with biopsy-proven myocarditis, 30 had NCV. Endomyocardial samples were retrospectively investigated with immunohistochemistry for toll-like receptor 4 (TLR4) and real-time polymerase chain reaction (PCR) for viral genomes. Serum samples were processed for anti-heart autoantibodies (Abs), IL-1β, IL-6, IL-8, tumour necrosis factor (TNF)-α. Identification of an immunologic pathway (including virus-negativity, TLR4-, and Ab-positivity) was followed by immunosuppression. Myocarditis-NCV cohort was followed for 6 months with 2D-echo and/or cardiac magnetic resonance and compared with 60 Myocarditis patients and 30 controls. Increase in left ventricular ejection fraction ≥10% was classified as response to therapy. Control endomyocardial biopsy followed the end of treatment. Twenty-six Myocarditis-NCV patients presented with heart failure; four with electrical instability. Cause of Myocarditis-NCV included infectious agents (10%) and immune-mediated causes (chest trauma 3%; drug hypersensitivity 7%; hypereosinophilic syndrome 3%; primary autoimmune diseases 33%, idiopathic 44%). Abs were positive in immune-mediated Myocarditis-NCV and virus-negative Myocarditis; Myocarditis-NCV patients with Ab+ presented autoreactivity in vessel walls. Toll-like receptor 4 was overexpressed in immune-mediated forms and poorly detectable in viral. Interleukin-1β was significantly higher in Myocarditis-NCV than Myocarditis, the former presenting 24% in-hospital mortality compared with 1.5% of Myocarditis cohort. Immunosuppression induced improvement of cardiac function in 88% of Myocarditis-NCV and 86% of virus-negative Myocarditis patients.

**Conclusion:**

Necrotizing coronary vasculitis is histologically detectable in 1.5% of Myocarditis. Necrotizing coronary vasculitis includes viral and immune-mediated causes. Intra-hospital mortality is 24%. The immunologic pathway is associated with beneficial response to immunosuppression.

## Graphical Abstract

**Figure:**
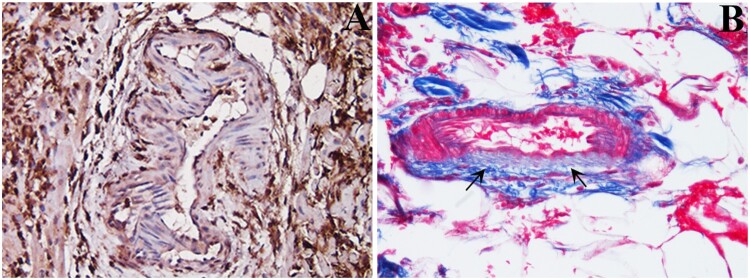

**See page 1618 for the editorial comment on this article (doi: 10.1093/eurheartj/ehab024)**

## Introduction

Myocarditis is an inflammatory disease of the heart frequently resulting from viral infections that cause direct cardiac damage and/or post-viral immune-mediated responses.^1^ Beyond infections, myocarditis can be caused by a large variety of autoimmune disorders, drugs, and toxins.^2^ It is an important heart-specific inflammatory entity causing heart failure, chest pain, unexplained arrhythmias, and sudden death. Prognosis in myocarditis patients depends on the underlying aetiology.^3^ Rarely, myocarditis is associated with necrotizing vasculitis of intramural vessels.

Aim of this retrospective study is to report incidence, cause, and outcome based on the underlying condition and its specific treatment.

## Methods

### Study population

At our institution from January 1983 up to July 2019, 5180 patients underwent an endomyocardial biopsy (EBM) because of suspected cardiomyopathy or myocarditis. A histological diagnosis of myocarditis was obtained in 37% of cases (1916) on the basis of the current definition.^4^ Among them, a necrotizing coronary vasculitis (NCV), defined as associated inflammatory infiltration with necrosis of intramural vessel wall, was documented in 30 patients (1.5%; 12 females and 18 males, with mean age of 47.7 years ± 15). All patients included in the study presented a severe cardiac compromise and at histology extensive myocarditis with at least two intramural arterioles or small arteries involved. This cohort [myocarditis-associated necrotizing coronary vasculitis (Myocarditis-NCV) patients] represents our study population. The study was approved by the local Ethics Committee and patients gave their informed consent.

Endomyocardial samples from all patients were retrospectively studied with histology, immunohistochemistry including myocardial expression of toll-like receptor 4 (TLR4) and real-time PCR for viral genomes. Serum samples were processed for anti-heart autoantibodies (Abs) and inflammatory cytokine profile. Myocarditis-NCV patients, defined as those presenting at histology CD3-positive T-lymphocytes ≥7 cells/mm^2^ associated to necrosis of adjacent myocytes with lymphocytic wall infiltration and necrosis of ≥2 arterioles or small coronary arteries, were compared with a group of 60 consecutive patients with virus-positive and virus-negative lymphocytic myocarditis and a control group of 30 patients with mitral stenosis and normal left ventricular (LV) size and function, undergoing surgical repair.

### Clinical studies

Clinical assessment, resting electrocardiogram (ECG), Holter monitoring, and 2D-echocardiography were performed at baseline in all patients. Cardiac catheterization, angiography, and left or biventricular EBM were also performed, after patients’ informed consent.

Echocardiographic parameters were determined according to established criteria.^5^ Cardiac magnetic resonance (CMR) imaging with late gadolinium enhancement was performed in 45% of cases, because clinical conditions were not compatible with the CMR or because the patients were evaluated before 2000. Functional and morphological data were analysed according to the Lake Louise criteria.^6^

All patients received conventional medical treatment for heart failure and arrhythmias.^7^ In addition, immunosuppressive therapy, including 1 mg/kg prednisone daily for 4 weeks followed by 0.33 mg/kg daily for 5 months and 2 mg/kg azathioprine daily for 6 months, was performed in case of virus-negative inflammatory cardiomyopathy or identification of an immunologic pathway (virus-negative, TLR4-positive, anti-heart Ab-positive).^8^ In case of specific primary autoimmune disorders (i.e. Systemic Lupus Erythematosus, Churg-Strauss syndrome, Takayasu arteritis and Giant cell myocarditis) other immunosuppressive regimens (cyclophosphamide, high-dose immunoglobulins, anakinra) were adopted. Patients were followed for 6 months and absolute increase of ≥10% in the left ventricular ejection fraction (LVEF) was classified as response to therapy.

Endomyocardial biopsies (3–6 fragments) were drawn in the septal–apical region of left or both ventricles.^9^ Samples were either fixed in formalin and paraffin-embedded for histopathology and immunohistochemistry or snap frozen for molecular biology. Histologic diagnosis of myocarditis was performed according to the Dallas criteria.^10^ For the phenotypic characterization of the inflammatory infiltrates, immunohistochemistry for Cluster of Differentiation (CD)3, CD20, CD43, CD45RO, and CD68 was performed (all Dako, Carpinteria, CA, USA). The presence of an inflammatory infiltrate ≥14 leucocytes/mm^2^ including up to 4 monocytes/mm^2^, with the presence of CD3-positive T-lymphocytes ≥7 cells/mm^2^ associated with evidence of degeneration and/or necrosis of the adjacent cardiomyocytes, was considered diagnostic for myocarditis.^4^ The number of CD3-positive cells was manually counted using a tally counter on high power field (×400) scanning the entire slide. The area of tissue samples was measured by means of a computerized system (Imaging Software/NIS-Elements AR 4.30, Nikon Instruments Inc., Melville, NY, USA). The number of CD3-positive cells was expressed as number of cells per square millimetre. Morphometric evaluation was performed by a pathologist blinded to clinical data. Moreover, it was determined the myocardial expression of TLR4, as already described.^11^ The endothelial cells of intramural vessel wall were identified by means of CD31 antibody (Monoclonal Mouse Anti-Human CD31, Endothelial cells, Dako Denmark A/S, 1:10).

In all patients at baseline, a real-time PCR analysis^12^ for the most common cardiotropic viruses (adenovirus, enterovirus, influenza A and B viruses, Epstein–Barr virus, Parvovirus B19 (PVB19), Hepatitis C virus, Cytomegalovirus, Human Herpes Virus 6, Herpes Simplex virus A and B) was performed.

### Serum studies

Serum samples were processed for anti-heart Abs and inflammatory cytokine profile including assessment of IL-1β, IL-6, IL-8, tumour necrosis factor (TNF)-α (ELLA assay).^13^ The presence of circulating anti-heart Abs was evaluated by indirect immunofluorescence as previously described.^14^ ^,^ ^15^ Briefly, patient sera were tested with a substrate of 4‐μm‐thick unfixed cryostat sections of human atrium and intercostal skeletal muscle of blood group O patients. The intensity of immunofluorescence of the positive control (known positive serum) at 1/40 dilution was used as the cut-off point for positivity. Omission of the patient serum and known negative serum was included in every assay (negative controls).

### Follow-up

Clinical assessment, resting ECG, Holter monitoring, and 2D-echocardiography were performed at baseline, weekly during the 1st month, every 4 weeks for the subsequent 5 months. Cardiac magnetic resonance was repeated in 45% of cases at the end of the study. All patients had a control biopsy at the end of immunosuppressive therapy. Control histology, immunohistochemistry for inflammatory cells, and TLR4 were obtained. Serum control studies for anti-heart antibodies and quantification of inflammatory cytokines were also undertaken.

### Statistical analysis

Quantitative measurements are expressed as mean ± SD. A value of *P < *0.05 was considered as significant. Categorical data were presented as absolute frequencies and per cent values. Difference between two groups was determined by unpaired *t*-test for continuous variables and Fisher’s exact test for categorical variables. Continuous variables are normally distributed. Multiple group comparisons were obtained for continuous variables with analysis of variance (ANOVA) and for categorical variables with χ^2^ test. *Post-hoc* analysis was performed using Bonferroni correction for continuous variables tested with ANOVA. The univariate and multivariable relationship among demographic, clinical, and echocardiographic data and presence of NCV and in-hospital mortality were also assessed.

## Results

### Myocarditis-necrotizing coronary vasculitis patients

An NCV has been histologically detected in 30 out of 1916 patients (1.5%) with myocarditis. No complications resulted from EBM even when applied to a severely compromised left ventricle.

#### Clinical manifestation and investigations

Twenty-six (87%) Myocarditis-NCV patients presented with heart failure or cardiogenic shock; 13% (*n* = 4) with electrical instability. No differences were registered in terms of age and gender. Time to symptoms onset was variable from few days to some weeks manifesting with acute cardiogenic shock or progressive cardiac deterioration. Impairment of cardiac function was found in nearly 87% of patients, often requiring inotropic drugs and in 11 patients mechanical circulatory support (such as extracorporeal membrane oxygenation, ECMO). Of the latter, 64% had an LVEF ≤25% and LV end-diastolic diameter (LVEDD) ≥60 mm. Two-dimensional echocardiography was performed in all patients at baseline and during follow-up. Cardiac magnetic resonance was performed in 45% of cases, because clinical conditions were not compatible with the CMR or because the patients were evaluated before 2000; of these patients, Lake Louise criteria were suggestive of myocarditis in 78% of cases. Cardiac magnetic resonance did not find qualitative signal abnormalities suggesting the presence of NCV.

Coronary vessels were normal in all patients, left ventriculography showed the presence of thrombi at the LV apex in eight subjects and small aneurysms in LV apical and posterior segments in nine cases.

High-sensitivity Cardiac Troponin was more elevated in Myocarditis-NCV (0.32 ± 0.2 μg/L) than Myocarditis cohort (0.1 ± 0.14 μg/L) (*P* < 0.01).

#### Aetiology

Myocarditis-NCV was infectious in three patients and the identified agents on cardiac tissue were Human Herpes Virus 2 (HHV2), Epstein–Barr Virus and in one case, a fatal PVB19-*Toxoplasma gondii* co-infection in an HIV-positive patient. In three patients, Myocarditis-NCV was auto-reactive and caused by blunt chest trauma in a 41-year-old football player and drug hypersensitivity in the other two patients, one of whom was under treatment with clozapine 250 mg/d for schizophrenia and the other with clomipramine 150 mg/d for depression; all these patients developed cardiogenic shock requiring inotropic support and ECMO. One patient had a hypereosinophilic syndrome. In 33% of patients, Myocarditis-NCV was associated to a primary autoimmune disorder including polyarteritis nodosa (*n* = 1); systemic lupus erythematosus (*n* = 2); coeliac disease (*n* = 1); granulomatous polyangiitis (*n* = 1); Churg-Strauss syndrome (*n* = 1), Takayasu arteritis (*n* = 1); and giant cells myocarditis (*n* = 3). This cohort presented with severe clinical impairment presenting as restrictive cardiomyopathy, LV aneurysm and/or thrombosis, dilated cardiomyopathy (DCM), or cardiogenic shock. In the remaining 44% cases (*n* = 13), Myocarditis-NCV was classified as idiopathic.

#### Endomyocardial biopsies and immunologic profile

**Figure 1:**
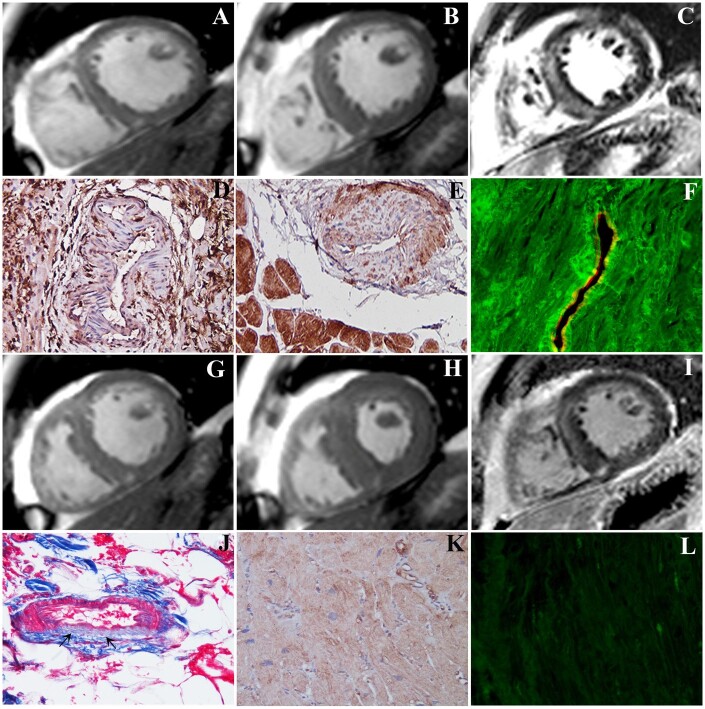
Immune-mediated myocarditis-associated necrotizing coronary vasculitis responding to immunosuppressive therapy. (*A–C*) Short-axis cardiac magnetic resonance showing remarkably dilated (*A*, EDV/BSA 124,7 mL/S2) and hypokinetic (*B*, ejection fraction 23%,) left ventricle with extensive subepicardial late gadolinium enhancement (*C*) suggesting severe myocarditis. (*D*) Severe lymphocytic myocarditis with necrotizing vasculitis of an intramural coronary artery (immunohistochemistry with CD45Ro, 200×). (*E*) Necrotizing coronary vasculitis with overexpression of toll-like receptor 4 in cardiomyocytes and vascular smooth muscle cells suggesting an immune-mediated mechanism of damage (immunoperoxidase for toll-like receptor 4, 200×). (*F*) Positive anti-heart antibodies (FITC green fluorescence) with autoreactivity for vessel wall, as showed by the co-localization with CD 31 antigen (TRITC red immunostaining), 400×. (*G–I*) Control cardiac magnetic resonance after immunosuppressive therapy showing normalization of left ventricular end-diastolic dimension (*G*, EDV/BSA 86,5 mL/S2), contractility (*H*, ejection fraction rising from 23% to 52%) and reduction with attenuation of late gadolinium enhancement (*I*). (*J–L*) Control biopsy after 6-month immunosuppressive therapy showing healed myocarditis with reparative fibrosis of vessel wall (*J*, arrows, Masson trichrome, 200×), reduction of toll-like receptor 4 immunoreactivity (*K*, immunoperoxidase for toll-like receptor 4, 200× magnification), and negativity of anti-heart antibodies (*L*, indirect immunofluorescence).

In all Myocarditis-NCV patients, histology showed extensive inflammatory infiltrates associated to cell necrosis (*Figure 1D*). Coronary intramural vessels were similarly infiltrated with necrosis of their wall (*Figure 1D*).

**Figure 2:**
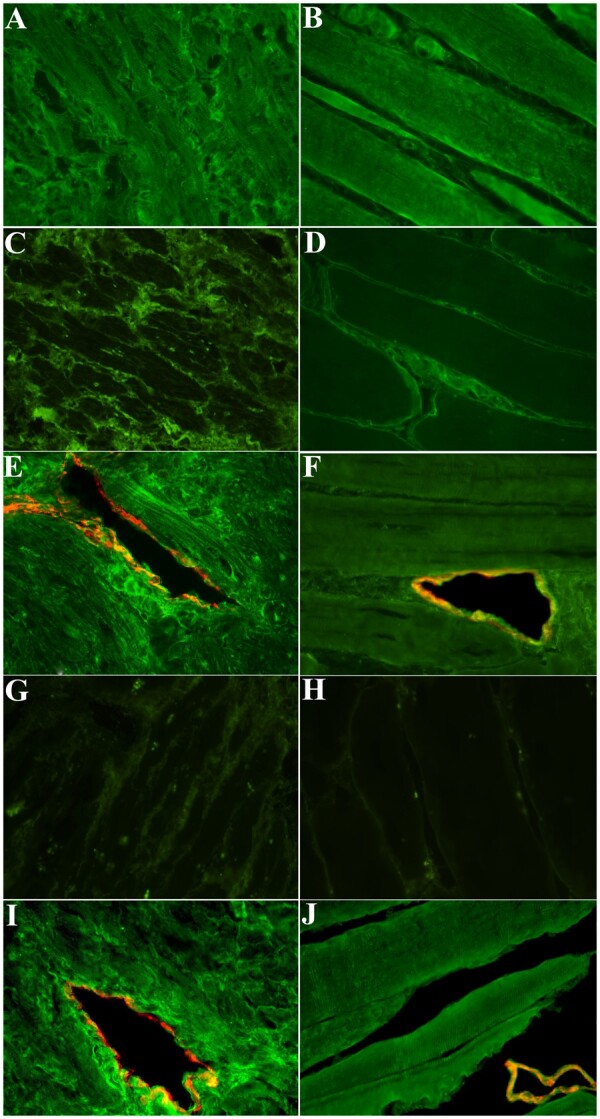
Anti-heart autoantibodies characterization: evidence for partially organ-specific pattern in patients with Myocarditis-necrotizing coronary vasculitis. (*A*) Positive partially organ-specific (fine striational) pattern of anti-heart autoantibodies on human heart (FITC green fluorescence, 400×) in the serum of a patient with Myocarditis-necrotizing coronary vasculitis. (*B*) Weakly positive staining for anti-heart autoantibodies on skeletal muscle (FITC green fluorescence, 400×) in the serum of the same patient of *A*. (*C*) Anti-heart autoantibody-negative control serum on human heart; no cardiomyocyte staining is present (FITC green fluorescence, 400×). (*D*) Anti-heart autoantibody-negative control serum on human skeletal muscle; no myocyte staining is present (FITC green fluorescence, 400×). (*E*) Positive serum for anti-heart and anti-endothelial cells autoantibodies on human heart (FITC green fluorescence, 400×) in a patient with Myocarditis-necrotizing coronary vasculitis. (*F*) Positive serum for anti-heart and anti-endothelial cells autoantibodies on skeletal muscle (FITC green fluorescence, 400×) in a patient with Myocarditis-necrotizing coronary vasculitis. (*G*) Negative control serum for fluoresceinated secondary antibody on human heart (FITC green fluorescence, 400×). (*H*) Negative control serum for fluoresceinated secondary antibody on skeletal muscle (FITC green fluorescence, 400×). (*I*) Positive control serum for anti-heart and anti-endothelial cell autoantibodies on human heart (FITC green fluorescence, 400×). (*J*) Positive control serum for anti-heart and anti-endothelial cell autoantibodies on human skeletal muscle (FITC green fluorescence, 400×).

Myocarditis-NCV was classified as immune-mediated when anti-heart Abs were positive, viruses in cardiac tissue were absent, myocardial TLR-4 was overexpressed and inflammatory cytokines (IL-1β, IL-6, IL-8, TNF-α) in serum samples were elevated. In particular, cardiac Abs were present in 27 (90%) of myocarditis-NCV patients with partially organ-specific pattern of staining, consisting in a strongly positive fine striational pattern on human heart tissue and a weak positive fine striational pattern on human skeletal muscle.^14^ Interestingly, in patients in whom anti-heart Abs were positive, the immunofluorescence was extended to intramural coronary vessels’ wall, as confirmed by co-localization for the endothelial antigen CD31 (*Figures 1F* and 2) also TLR4 was overexpressed both in cardiomyocytes and in necrotized vessels (*Figure 1E*).

#### Management and follow-up

All patients received conventional medical treatment for heart failure and arrhythmias. Immune-mediated Myocarditis-NCV patients were treated with immunosuppression including 1 mg/kg prednisone daily for 4 weeks followed by 0.33 mg/kg daily for 5 months, and 2 mg/kg azathioprine daily for 6 months according to the Tailored Immosuppression in Inflammatory Cardiomyopathy (TIMIC) trial.^8^ The auto-reactive Myocarditis-NCV group was treated with high-dose steroids and withdrawal of the culprit agent in case of drug hypersensitivity and subsequently showed full recovery of LV function. In the giant cell Myocarditis-NCV group (*n* = 3), an aggressive immunosuppressive treatment with high-dose steroids and azathioprine was administered (prednisolone iv 7 mg/kg for 3 days, followed by prednisone 1.5 mg/kg/d orally for 2 weeks tapered to 1 mg/kg/d for 4 weeks; then prednisone was reduced to 0.33 mg/kg/d and azathioprine 2 mg/kg/d was included in the treatment; after 6 months, steroids were tapered and withdrawn; azathioprine from the 6th month was reduced to 1 mg/kg/d); in two of these patients, cardiac function was totally recovered. Patients with primary autoimmune disorder received immunosuppressive therapy including steroids, azathioprine, and cyclophosphamide with complete resolution of myocarditis. One patient with virus-negative inflammatory cardiomyopathy and decompensated type 2 diabetes mellitus was treated with high-dose immunoglobulin and then with IL-1β inhibitor (anakinra) with improvement of ventricular function.

Two patients with immune-mediated Myocarditis-NCV were not treated with immunosuppression because death occurred because of cardiogenic shock or ventricular fibrillation.

Immunosuppression resulted in an improvement of cardiac function in 22 (88%) out of 25 Myocarditis-NCV-treated patients. None of the patients on immunosuppression had major drug-related side effects requiring therapy withdrawal; minor adverse reaction as increased body weight, glucose blood level elevation and fluid retention requiring diet, oral anti-diabetic drugs or insulin administration, and diuretic dose adjustment were reported in 30% of cases.

**Figure 3:**
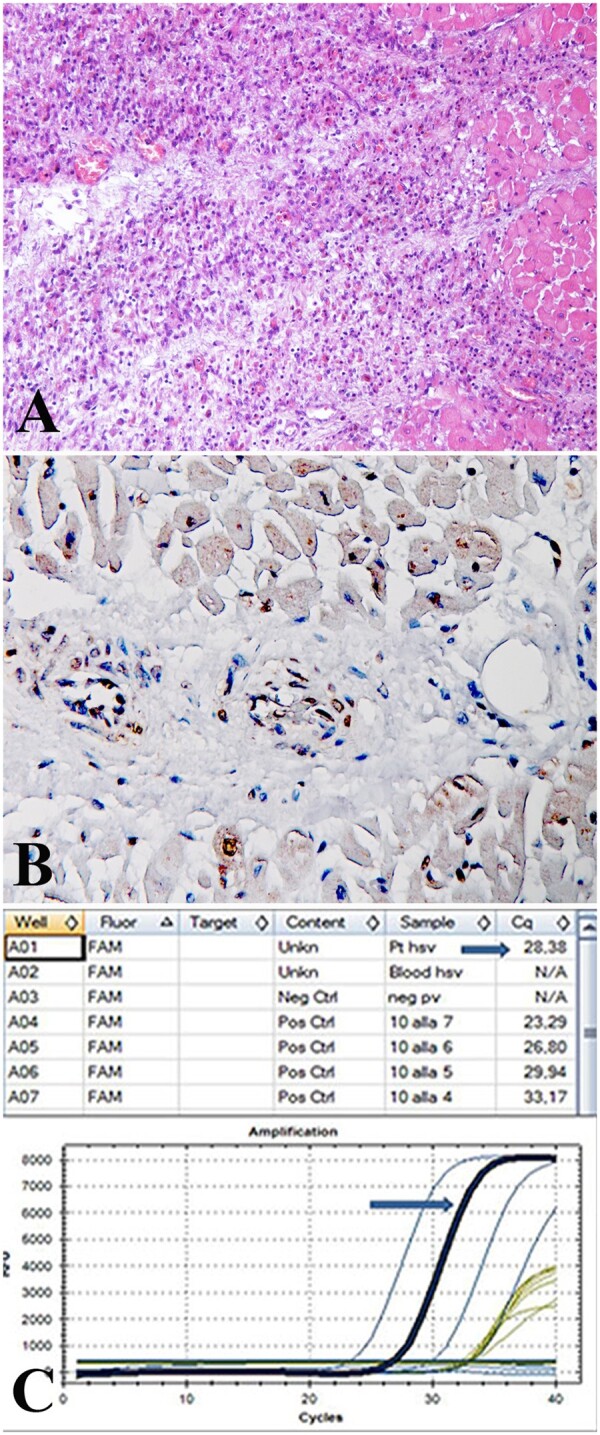
Post-mortem histologic and molecular study in a 33-year woman died because of infectious myocarditis with necrotizing coronary vasculitis. (*A*) Massive lymphocytic myocarditis with extensive cell necrosis (H&E 100×). (*B*) Necrotizing intramural coronary vasculitis with no expression of toll-like receptor 4 suggesting an infectious cause (immunohistochemistry for toll-like receptor 4, 200×). (*C*) Real-time PCR indicating a myocardial infection by Human Herpes Virus 2.

Death occurred during hospitalization in seven patients with Myocarditis-NCV because of cardiogenic shock or cardiac arrest. In-hospital death (24%) was remarkably higher than that (1.5%) of Myocarditis cohort. Regarding aetiology, death occurred in the HIV-positive patient with PB19V-*T. gondii* co-infection, in the patient with HHV2 related-myocarditis because the viral infection caused a severe Myocarditis-NCV (*Figure 3*) and infiltration of cardiac conduction tissue and ganglia inducing ventricular fibrillation, in the patient with hypereosinophilic syndrome, in one patient with giant cells and in three patients with immune-mediated Myocarditis-NCV.

Control biopsy in patients receiving immunosuppressive therapy showed progression of inflammatory disease to healed myocarditis and vasculitis with disappearance of inflammatory cells, halting of tissue necrosis and myocardial and vessel reparative fibrosis, attenuated expression of TLR4 and negative anti-heart antibodies (*Figure 1J–L*).

In patients with virus-negative Myocarditis, immunosuppression induced an improvement of cardiac contractility in 86% of cases.

### Comparison between Myocarditis-necrotizing coronary vasculitis patients, M patients and controls

Baseline clinical parameters and immunological pattern of Myocarditis-NCV patients compared with Myocarditis patients and normal controls are shown in *Table 1* (overall *P*-values). Supplementary material online, *Table* shows the *P*-value for each comparison.

**Table 1:** Baseline clinical parameters and immunological pattern of Myocarditis-necrotizing coronary vasculitis patients compared with Myocarditis patients and normal controls

Patients’ characteristics	Myocarditis-NCV pts, *n* = 30	Myocarditis pts, *n* = 60	Controls, *n* = 60	**Overall *P-*values** ^a^
Age (years)	47.7 ± 15.0	49.3 ± 14.5	48 ± 15.8	0.864
Sex	18 M (60%)	37 M (61%)	19 M (63%)	0.198
	12F (40%)	23F (39%)	11F (37%)	
Clinical manifestation				
Heart failure	26 (87%)	50 (83%)^c^	0 (0%)^d^	0.000
Electrical instability	4 (13%)	10 (17%)^c^	0 (0%)^d^	0.064
2D-echocardiography				
LVEDD (mm)	57.2 ± 8.6	58.7 ± 8.1^c^	47.3 ± 11.9^d^	0.000
LVESV (mL/m^2^)	105.5 ± 32.7	105.4 ± 33.1^c^	82.7 ± 18.7^d^	0.001
LVESV (mL/m^2^)	76.8 ± 33.8	70.4 ± 33.8^c^	31.7 ± 6^d^	0.000
LVEF (%)	30 ± 0.1	34 ± 1.3^c^	59 ± 0.7^d^	0.000
MWT	10.5 ± 1.9	11.4 ± 2.9^c^	9.8 ± 1.1	0.008
Immunological pattern				
Circulating IL-1b (pg/mL)	2.82 ± 8.0^b^	0.57 ± 0.34	0.35 ± 0.24^d^	0.021
Circulating IL-8 (pg/mL)	12.41 ± 18.4	8.42 ± 4.6	3.27 ± 0.7^d^	0.002
Myocardial TLR4	3.21 ± 0.65^b^	1.42 ± 1.42^c^	0.06 ± 0.1^d^	0.000
Anti-heart abs positivity (partially organ-specific pattern)	27 (90%)	55 (92%)^c^	0 (0%)^d^	0.000
hs cTn (µg/L)	0.32 ± 0.2^b^	0.1 ± 0.14	0.012 ± 0.004^d^	0.000

Abs, autoantibodies; hs cTn, high-sensitivity Cardiac Troponin (nv < 0.014 µg/L); LVEDD, left ventricular end-diastolic diameter; LVEDV, left ventricular end-diastolic volume; LVEF, left ventricular ejection fraction; LVESV, left ventricular end-systolic volume; MWT, maximal wall thickness; TLR4, toll-like receptor 4.

a *P*-values referred to comparison between three groups.

b Referred to statistically significant difference between M-NCV and M groups.

c Referred to statistically significant difference between M-NCV and Control groups.

d Referred to statistically significant difference between M and Control groups. A *P*-value <0.05 was considered statistically significant.

Cardiac magnetic resonance imaging did not detect any qualitative difference between Myocarditis-necrotizing coronary vasculitis and Myocarditis patients.

Anti-heart Abs were positive in all the immune-mediated Myocarditis-NCV (*n* = 27), and virus-negative Myocarditis (*n* = 55), while were negative in viral forms of myocarditis and in normal controls.

Myocardial expression of TLR4 was increased in the immune-mediated forms and poorly detectable in the viral forms and in the control group (who did not show any sign of myocardial inflammation at histology). Circulating interleukins 1-β and IL-8 were higher in patients with Myocarditis-NCV (2.82 ± 8.0 and 12.41 ± 18.4 pg/mL, respectively) in comparison with virus-negative Myocarditis patients (0.57 ± 0.34 and 8.42 ± 4.6 pg/mL, respectively) and control group (0.35 ± 0.24 and 3.27 ± 0.7 pg/mL, respectively). IL-6 and TNF-α did not show any statistical relevant difference between two groups (Myocarditis-NCV vs. Myocarditis).

Fisher's test showed a significant association between in-hospital mortality and the presence of NCV (*P* = 0.002).

In the univariate analysis, the variables that were significantly associated with in-hospital mortality among all Myocarditis patients with and without NCV were female gender (OR 4.8, 95% CI: 1.1–22, *P* = 0.041), and presence of coronary necrotizing vasculitis (OR 17.9, 95% CI: 2.1–154, *P* = 0.008) (*Table 2*). At multivariable analysis presence of coronary necrotizing vasculitis (OR 15.4, 95% CI: 0.7–71, *P* = 0.014), was confirmed to significantly predict in-hospital mortality.

**Table 2:** Association between in-hospital death and myocarditis with necrotizing coronary vasculitis in univariate and multivariable analysis

Variable	Univariate	*P*-value	Multivariable	***P*-value** ^a^
NCV	17.9 (2.1–154)	0.008	15.4 (0.7–71)	0.014
Age	1.04 (0.98–1.1)	0.166		
Female sex	4.8 (1.1–22)	0.041	3.7 (0.7–17.18)	0.116
Heart failure	1.32 (0.15–11.6)	0.803		
Arrhythmic instability	1.32 (0.15–11.6)	0.803		
LVEF	0.97 (0.92–1.03)	0.357		

LVEF, left ventricular ejection fraction; NCV, necrotizing coronary vasculitis.

a *P*-value referred to comparison between three groups; *P-*value <0.05 was considered statistically significant.

A limitation for the validity of statistical analysis is that confidence interval for NCV is wide and it is likely related to the limited sample size.

## Discussion

Myocarditis is a major cause of heart muscle disease concurring approximately to one-third of diagnoses obtained from EBM studies.^9^ Rarely it may complicate with inflammation of intramural vessels causing severe impairment of cardiac function and/or enhancement of electrical instability leading to increased in-hospital mortality. Its knowledge is limited to occasional, single case reports^16–19^ while extensive studies on incidence, cause, and outcome are not reported in literature. Myocarditis-NCV was recognized in our study in 1.5% (30 cases) of 1916 consecutive patients with a histological diagnosis of myocarditis. Diagnosis of myocarditis followed the Dallas criteria^10^ implemented by immunohistochemical characterization of the inflammatory cells. In-hospital mortality was 24% compared with 1.5% occurred in 60 consecutive patients with isolated acute myocarditis. Elevated Myocarditis-NCV mortality was not preventable by use of ventricular assist device and/or ICD implantation confirming its grim prognosis.

Myocarditis-NCV was not distinguishable from myocarditis alone by CMR and its identification can only be obtained by EBM. On the other hand, it is recognized^20^ that even diagnostic CMR sensitivity for myocarditis while is high (>90%) for infarct-like phenotype is limited (<50%) for cardiomyopathic and arrhythmic presentation. In addition, CMR cannot influence myocarditis treatment as it does not provide details on histological, immunohistochemical, and molecular characterization of myocardial tissue. All these considerations make EBM the ‘gold standard’ for diagnosis and treatment of myocarditis.^4^ ^,^ ^21^

In our study, LV biopsy has been revealed safe even in patients with severely compromised and electrically unstable ventricle being followed by no complications.

Regarding the cause of Myocarditis-NCV, infectious agents, auto-reactive mechanisms, and autoimmune diseases were mostly involved.

Poorly opposable myocardial viral infection as those represented by HHV2 and co-infection by *T. Gondii* and ParvoB19 were among the deadly infectious causes.

The most common pathogenetic instance for Myocarditis-NCV was, however, a virus-negative immune-mediated inflammation. This included autoreactivity to myocardial antigens released after a chest trauma or newly generated haptens by drugs, as clozapine^17^ and clomipramine^18^ administration. Primary autoimmune diseases like Systemic Lupus Erythematosus and Giant Cell myocarditis were among the most common specific entities while non-specific immune-mediated pathways were encountered in 13 cases. Severity of viral or non-viral myocardial and vessels inflammation suggests the occurrence of a cytokine storm. Indeed levels of interleukin 1β were more pronounced in patients with Myocarditis-NCV compared with isolated myocarditis.

It is remarkable that all cases with immune-mediated Myocarditis-NCV were virus-negative at myocardial PCR, had positive anti-heart Abs with partially organ-specific pattern, and showed overexpression of TLR4 at tissue immunohistochemistry. Particularly, the last aspect expresses tissue exposition of new antigens and the likely immunogenic origin of the inflammatory process.^11^

This pattern of differentiation with virus-induced Myocarditis-NCV is crucial for treatment particularly whenever, because of limited PCR panel, the viral agent is missed.

Indeed, 22 Myocarditis-NCV patients (88%) with immune-mediated pathway among 25 treated with immunosuppression responded to the treatment with improvement of ejection fraction (EF) ≥10% and 77% of them manifested a complete recovery of cardiac function (see *Figure 1*). Immunosuppression included a various combination of steroids with azathioprine, cyclophosphamide, and high-dose immunoglobulins. In a patient with diabetes mellitus, inhibitor of interleukin 1β (anakinra) was adopted. No major side effects from immunosuppression administration were registered.

Improvement or recovery of LV function was associated at control biopsy by disappearance of inflammatory infiltrates in the myocardium and intramural vessels that presented evidence of replacement fibrosis.

Therefore, Myocarditis-NCV clinically manifests as fulminant myocarditis, with severe haemodynamic compromise requiring aggressive inotropic support in the acute phase but with a beneficial response to immunosuppression.^22^

Finally, in patients with virus-negative Myocarditis, immunosuppression induced an improvement of cardiac contractility in 86% of cases. These results were comparable with those already reported in the TIMIC trial.^8^

## Conclusion

Necrotizing coronary vasculitis can be histologically detected in up to 1.5% of patients with myocarditis. Necrotizing coronary vasculitis includes viral, auto-reactive, and autoimmune causes. Intra-hospital mortality is remarkably higher than for patients with isolated myocarditis. Identification of an immunologic pathway is associated with a beneficial response to immunosuppression.

## Supplementary material

Supplementary material is available at *European Heart Journal* online.

## Funding

The European Project ERA-CVD ‘Transnational Research Projects on Cardiovascular Diseases’ (JTC 2016 IKDT-IGCM) and by Italian Ministry of Health ‘Ricerca corrente’ IRCCS Spallanzani.

**Conflict of interest:** none declared.

## Supplementary Material

ehaa973_Supplementary_Table
