# Supplementary material for: Myocarditis-associated necrotizing coronary vasculitis: incidence, cause, and outcome
Source: Eur Heart J. 2020 Dec 23;42(16):1609–17. doi: 10.1093/eurheartj/ehaa973 (PMC8088814; doi:10.1093/eurheartj/ehaa973)
Supplement: ehaa973_Supplementary_Table [file ehaa973_supplementary_table.docx]

**Supplementary Table.** Comparison for each variable among the different groups

| **Characteristics** | **p-values Myocarditis-NCV pts vs Myocarditis pts** | **p-values Myocarditis-NCV pts vs controls** | **p-values Myocarditis pts vs controls** | **Overall p values*** |
| --- | --- | --- | --- | --- |
| **Age (years)** | 1 | 1 | 1 | 0.864 |
| **Sex** | 0.100 | 0.791 | 0.183 | 0.198 |
| **Clinical manifestation** |  |  |  |  |
| *Heart failure* | 0.681 | 0.000 | 0.000 | 0.000 |
| *Electrical instability* | 0.681 | 0.038 | 0.018 | 0.064 |
| **2d-echocardiography** |  |  |  |  |
| LVEDD (mm) | 1 | 0.000 | 0.000 | 0.000 |
| LVEDV (ml/m2) | 1 | 0.006 | 0.002 | 0.001 |
| LVESV (ml/m2) | 0.830 | 0.000 | 0.000 | 0.000 |
| LVEF (%) | 0.148 | 0.000 | 0.000 | 0.000 |
| MWT (mm) | 0.225 | 0.832 | 0.008 | 0.008 |
| **Immunological Pattern** |  |  |  |  |
| Circulating IL-1b (pg/ml) | 0.035 | 0.048 | 1 | 0.021 |
| Circulating IL-8 (pg/ml) | 0.217 | 0.001 | 0.060 | 0.002 |
| Myocardial TLR4 | 0.000 | 0.000 | 0.000 | 0.000 |
| Antiheart abs positivity (partially organ specific) | 0.793 | 0.000 | 0.000 | 0.000 |
| **hs cTn (µg/l)** | 0.000 | 0.000 | 0.057 | 0.000 |

*p values referred to comparison between three groups. p value <0.05 was considered statistically significant. LVEDD = Left ventricular end-diastolic diameter, LVEDV = Left ventricular end-diastolic volume; LVESV = Left ventricular end-systolic volume; LVEF = Left ventricular ejection fraction; MWT = maximal wall thickness; TLR4 = Toll-like Receptor 4; Abs = autoantibodies; hs cTn = high-sensitivity Cardiac Troponin (nv < 0.014 µg/l).
